# A Preliminary Investigation of the Association Between Misophonia and Symptoms of Psychopathology and Personality Disorders

**DOI:** 10.3389/fpsyg.2020.519681

**Published:** 2021-01-14

**Authors:** Clair Cassiello-Robbins, Deepika Anand, Kibby McMahon, Jennifer Brout, Lisalynn Kelley, M. Zachary Rosenthal

**Affiliations:** ^1^Department of Psychiatry and Behavioral Sciences, Center for Misophonia and Emotion Regulation, Duke University Medical Center, Durham, NC, United States; ^2^Department of Psychology and Neuroscience, Duke University, Durham, NC, United States; ^3^International Misophonia Research Network, New York, NY, United States

**Keywords:** misophonia, psychopathology, personality disorders, mediation, anxiety

## Abstract

Misophonia is a condition characterized by defensive motivational system emotional responding to repetitive and personally relevant sounds (e.g., eating, sniffing). Preliminary research suggests misophonia may be associated with a range of psychiatric disorders, including personality disorders. However, very little research has used clinician-rated psychometrically validated diagnostic interviews when assessing the relationship between misophonia and psychopathology. The purpose of this study was to extend the early research in this area by examining the relationship between symptoms of misophonia and psychiatric diagnoses in a sample of community adults, using semi-structured diagnostic interviews. Results indicated higher misophonia symptoms were associated with more clinician-rated symptoms of personality disorders, but not other disorders. Anxiety partially mediated the relationship between personality disorder symptoms and misophonia. These results suggest misophonia may be associated with a range of psychiatric symptoms and highlight the role of anxiety in this poorly understood condition.

Misophonia (denoted as “hatred of sound”) recently has been recognized as a “complex neurobehavioral syndrome phenotypically characterized by heightened autonomic nervous system arousal and negative emotional reactivity…” (Brout et al., 2018) in response to certain repetitive and pattern based sounds. Individuals with misophonia report heightened sympathetic nervous system arousal and emotional distress in response to personally relevant sounds (e.g., sniffing, eating, tapping; Jastreboff and Jastreboff, 2001; Edelstein et al., 2013). Psychophysiological data indicate individuals with misophonia show heightened defensive motivational system responses to misophonic (e.g., eating, breathing) vs. aversive (e.g., baby cry, person screaming) sounds, suggesting greater emotional reactivity in response to misophonic sounds (Kumar et al., 2017). Research primarily using self-report and interview data indicate a wide range of emotions can be experienced in response to misophonic sounds including anger, disgust, and anxiety (Edelstein et al., 2013; Schröder et al., 2017).

Beyond discrete emotional responses, preliminary research suggests misophonia may co-occur with a wide range of psychiatric disorders. Much of this early research relied on convenience samples and/or self-report methods. For example, studies exploring the relationship between misophonia and psychiatric diagnoses using online surveys and other self-report methods point to the possibility that higher misophonia symptom severity may be associated with symptoms or a diagnosis of attention-deficit hyperactivity disorder (ADHD), obsessive-compulsive disorder (OCD), eating disorders, obsessive-compulsive personality disorder (OCPD), anxiety, post-traumatic stress disorder, and depression (Wu et al., 2014; Rouw and Erfanian, 2018). While self-report data are valuable, clinician-rated interviews are typically preferred as part of the assessment when diagnosing psychiatric disorders (Klonsky and Oltmanns, 2002). Two studies investigating misophonia have used the Structured Clinical Interview for the Diagnostic and Statistical Manual of Mental Disorders—4th edition (DSM-IV) personality disorders (SCID-II). Natalini et al. (2019) described three cases in which all patients met criteria for OCPD, two met criteria for co-occurring borderline personality disorder (BPD), and one met criteria for co-occurring avoidant personality disorder (APD). Schröder et al. (2013) reported 52.4% of participants with misophonia (*N* = 42) met criteria for OCPD. In addition to the SCID-II, Schröder et al. (2013) used an unspecified psychiatric interview and reported 7.1% of their sample met criteria for mood disorders, 4.8% for ADHD, 2.4% for panic disorder, and 2.4% for OCD. Using the SCID for DSM, 5th edition (DSM-5), Frank and McKay (2019) reported diagnoses in their sample (*N* = 18) including depressive (22%), alcohol use (11%), panic (6%), generalized anxiety (17%), and social anxiety (11%) disorders as well as specific phobia (11%), agoraphobia (11%), excoriation (6%), hypersomnolence (11%), and ADHD (6%). Jager et al. (2020) used the MINI-International Neuropsychiatric Interview and SCID-II to assess adults reporting misophonia. These researchers indicated 72% of their sample did not have a comorbid psychiatric disorder. Participants with comorbid disorders met criteria for mood disorders (10.1%), anxiety disorders (9%), autism spectrum disorder (2.4%), somatoform disorder (1/4%), substance use disorder (1.6%), impulse control disorder (2.1%), tic disorder (1.6%), ADHD (5.4%), “other” (eating, psychotic, neurocognitive disorders; 1.4%), and personality disorders (5%). Taken together, these preliminary data suggest some, but not all, adults with misophonia report symptoms that co-occur with a wide range of psychopathology.

In addition to identifying the diagnoses that co-occur with misophonia, understanding the emotional patterns linking misophonia with other disorders would allow clinicians to choose interventions designed to specifically target these presentations. Previous research provides preliminary evidence for specific emotional responses associated with misophonia. For example, anger may lead to notable impairments and daily functioning (Schröder et al., 2013). Wu et al. (2014) reported anxiety mediated the relationship between misophonia and anger outbursts, raising the question as to whether other emotions underlie the relationship between misophonia and anger. Thus, it is possible the anger reported in misophonia may be secondary to anxiety experienced in response to trigger cues. However, this study was preliminary and the mediating role of other emotions, such as depression, has not been explored. Thus, there is a need for continued examining of the emotional patterns associated with misophonia and psychiatric disorders.

Currently there is limited work examining the relationship of misophonia with extant psychiatric diagnoses and exploring the emotions mediating these relationships. In order to characterize the relationship between misophonia and psychiatric disorders, it is essential to use psychometrically validated semi-structured diagnostic interviews. To the best of our knowledge, few studies have used a validated instrument to assess both personality and non-personality related psychopathology in the same sample. The purpose of this paper is to continue examining these relationships using clinician-rated interviews (i.e., SCID) in order to provide preliminary data characterizing the relationships between misophonia and psychopathology in a community sample of adults. Specifically, we investigated the association of misophonia symptoms with symptoms of psychopathology (e.g., depression, anxiety) and explored whether high vs. low misophonia symptoms were associated with psychiatric disorders including anxiety, mood, eating, and personality disorders. We hypothesized (1) high levels of misophonia symptoms would be associated with higher levels of depression and anxiety, (2) high misophonia would be associated with anxiety, mood, eating, and personality disorders compared to low misophonia, and (3) consistent with Wu et al. (2014), anxiety would mediate the relationship between misophonia and symptoms of psychopathology.

## Methods

### Participants

Forty-nine participants were drawn from a larger study examining the relationship between symptoms of psychopathology and sensory processing dysfunction in adults. Participants were included in the current analyses if they completed the Misophonia Questionnaire (MQ; Wu et al., 2014), which was an addition to the larger study (see section “Procedures”). Participants were excluded from the study if they were under age 18 or met criteria for current mania or psychotic disorder. Apart from these criteria, participants were not required to meet specific diagnostic or demographic criteria because the purpose of the larger study from which these data were derived was to gather information about emotion regulation and sensory processing from a general community sample.

Participants primarily identified as female (*n* = 42), non-Hispanic (*n* = 46), and White (*n* = 32). The average age of participants included in these analyses was 27.02 years (*SD* = 8.75). The majority of participants indicated they were single, never married (*n* = 35), had completed some college (*n* = 18), and had a salary range of $0–$10,000 (*n* = 27).

### Measures

#### Misophonia Questionnaire (MQ; Wu et al., 2014)

The MQ is a 17-item, three-part, questionnaire that evaluates the presence of misophonia symptoms, emotions, and behaviors associated with misophonic reactions, and the overall severity of an individual’s sound sensitivity. The first two subscales are rated on a scale of 0 (not at all true) to 4 (always true). These two parts are summed to produce a total score ranging from 0 to 68. The severity subscale is a single item that asks an individual to provide a rating of their sound sensitivity on a scale from 0 (no sound sensitivities) to 15 (very severe sound sensitivities). In its initial validation the MQ showed good internal consistency (α = 0.86–0.89). Reliability in this study was also good (α = 0.93).

#### Beck Depression Inventory-II (BDI; Beck et al., 1996)

This 21-item self-report measure assesses depressive symptoms experienced over the past week. Items consist of clusters of a range of severity for a given symptom and participants select the option that applies best to them over the past 2 weeks. Each item is score from 0 to 3 and items are summed to produce a total score. Higher total scores indicate greater severity of depression symptoms. This measure had sound internal consistency (α = 0.82) in its initial validation study and in the current study (α = 0.88).

#### Beck Anxiety Inventory (BAI; Beck and Steer, 1990)

The BAI is a 21-item self-report measure of anxiety symptoms. Participants are asked to indicate how much a given symptom (e.g., nervous) has bothered them during the past week on a scale of 0 (not at all) to 3 (severely—it bothered me a lot). Items are summed to produce a total score (range 0–63) with higher scores indicating greater symptom severity. This measure has demonstrated good reliability in clinical and non-clinical samples (0.92 and 0.93, respectively, Beck et al., 1996). It also showed good reliability in this study (α = 0.93).

#### Structured Clinical Interview for DSM-IV-I (SCID-I; First et al., 1995)

The SCID-I is a semi-structured clinical interview designed to assess the presence of DSM-IV Axis I disorders. In this study, current anxiety disorder was defined as currently meeting criteria for at least one of the follow diagnoses: panic disorder, agoraphobia, social phobia, specific phobia, obsessive-compulsive disorder, post-traumatic stress disorder, generalized anxiety disorder, anxiety due to general medical condition, substance-induced anxiety disorder, or anxiety disorder not otherwise specified (NOS). Current mood disorder consisted of meeting criteria for bipolar disorder I, bipolar disorder II, other bipolar disorder, major depressive disorder, dysthymic disorder, mood disorder due to general medical condition, or substance-induced mood disorder. Finally, current eating disorder was defined as meeting diagnostic criteria for anorexia nervosa, bulimia nervosa, binge eating disorder, and/or eating disorder NOS. Inter-rater reliability was assessed by a blind rater randomly rating 20% of SCID-I interviews from the parent study. Kappas ranged from 0.63 to 1.0, reflecting acceptable inter-rater reliability.

#### Structured Clinical Interview for DSM Disorders-II (SCID-II; First et al., 1995)

The SCID-II is a semi-structured clinical interview that assesses the presence of DSM-IV Axis-II (personality) disorders. Inter-rater reliability was assessed by a blind rater randomly rating 15% of SCID-II interviews from the parent study. Intraclass correlation coefficients ranged from 0.66 (Schizotypal) to 1.0 (Histrionic) indicating acceptable inter-rater reliability.

### Procedures

The Duke University Medical Center Institutional Review Board approved all procedures and all participants provided informed consent before beginning study procedures. All participants who provided consent completed study procedures. Recruitment for the larger study consisted of local list serve notices and clinic flyers in an academic medical center outpatient clinic. Interested participants contacted the clinic, completed a phone screen to ensure they did not meet exclusion criteria, and were scheduled for an in-person study visit. All procedures took place at the outpatient clinic. Participants completed interviews consisting of SCID-I and II (see section “Measures”), treatment history, and sensory processing dysfunction. Assessors were five study staff, comprised of three clinicians with a masters in social work, one doctoral student, and one research program leader; four assessors were female. All assessors were trained by observing the lead clinical assessor (a licensed clinical social worker) conduct three SCID interviews and then conducting a minimum of three interviews under observation.

Participants also completed a battery of self-report questionnaires regarding emotional functioning, sensory processing, and psychiatric symptoms. Finally, participants were debriefed, completed a relaxation exercise to reduce any unpleasant emotions associated with participation, and received a list of community treatment resources. See McMahon et al. (2019) for a detailed description of procedures.

### Data Analytic Plan

Data analyses were conducted in SPSS version 26. Correlational analyses examining the relationship between misophonia symptoms and self-reported anxiety and depression, as well as number of personality disorder (PD) symptoms on the SCID-II, were conducted using the MQ total score as a continuous measure. Analyses also examined differences in both self-report and clinician rated symptoms of psychopathology between individuals who reported high vs. low misophonia symptoms. To conduct these between-group analyses participants were divided into two groups (high and low misophonia symptoms) on the basis of their MQ total score. Participants with scores below 34 were considered to have low misophonia symptoms and those with scores above 34 were in the high misophonia group. The score of 34 was chosen because (a) there is no empirically derived or consensus standard for misophonia symptom severity on the MQ and (b) individuals with this score, or higher, would have an average score of 2 or higher across all the items on the MQ symptom and emotion subscales. Hedges g effect sizes, which include a correction for small samples, were used to examine the magnitude of the difference between high and low misophonia groups on continuous outcomes (BAI, BDI, MQ, number of PD symptoms). Categorical analyses were conducted using the high vs. low misophonia groups and the presence or absence of meeting criteria for anxiety, depressive, eating, and personality disorders.

**FIGURE 1:**
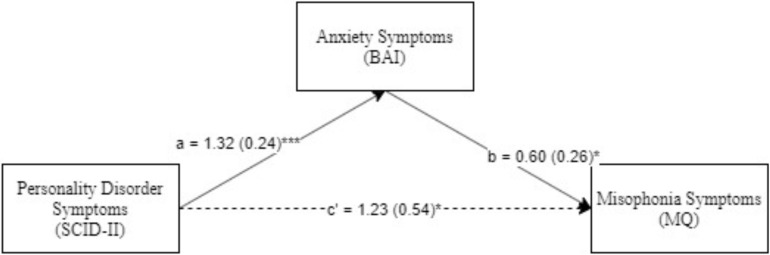
Personality disorder symptoms predicting mosophonia symptoms with anxiety symptoms as mediator. *p < 0.05, ***p< 0.001. Standard errors are in parentheses, solid lines represent significant indirect paths, a = unstandardized regression coefficient for the IV predicting the mediator, b = unstandardized regression coefficient for the mediator predicting the DV with IV and mediator in the model, c = unstandardized coefficient for the IV predicting the DV with the mediator in the model (direct effect). BAI, Beck anxiety inventory total score; MQ, Misphonia Questionaire total score; SCID-II, Structured clinical interview for DSM-IV (Axis II Disorders).

**FIGURE 2:**
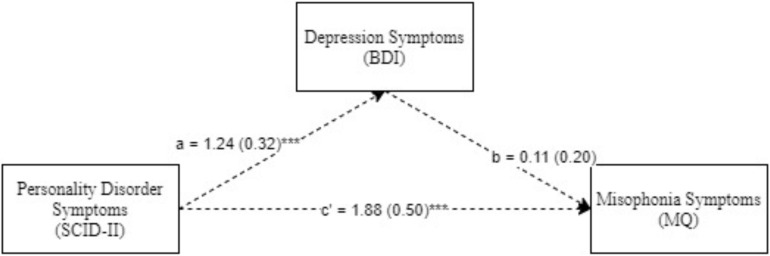
Personality disorder symptoms predicting mosophonia symptoms with anxiety symptoms as mediator. ***p< 0.001. Standard errors are in parentheses, solid lines represent significant indirect paths, a = unstandardized regression coefficient for the IV predicting the mediator, b = unstandardized regression coefficient for the mediator predicting the DV with IV and mediator in the model, c = unstandardized coefficient for the IV predicting the DV with the mediator in the model (direct effect). BAI, Beck anxiety inventory total score; MQ, Misphonia Questionaire total score; SCID-II, Structured clinical interview for DSM-IV (Axis II Disorders).

Next, we explored the mediating role of depression and anxiety in the relationship between clinician-rated psychiatric symptoms and misophonia. These models were explored only for diagnoses that were differentially associated with high vs. low misophonia. The models were examined using Model 4 in PROCESS, an SPSS macro for path-analysis based modeling (Hayes, 2018). We first explored single and then double (parallel) mediational models (Hayes, 2017). The single mediator models examined were, Model 1 (Figure 1): Psychiatric Symptoms (IV) → Anxiety Symptoms (BAI; Mediator) → Misophonia Symptoms (MQ; DV) and Model 2 (Figure 2): Psychiatric Symptoms (IV) → Depression Symptoms (BDI; Mediator) → Misophonia Symptoms (MQ; DV). A double (parallel) mediation model including both depression and anxiety symptoms as mediators was also explored. All possible indirect paths were tested in all models. Additionally, non-parametric bootstrapping was used to test the significance of indirect effects, in which the effect is interpreted as significant if 95% bias-corrected confidence intervals (CIs) for the effect do not include zero (Preacher and Hayes, 2004, 2008). Mediation analyses were based on 5,000 bootstrapped samples (as recommended by Hayes, 2009) using bias-corrected 95% CIs.

## Results

Shapiro-Wilk tests indicated all data were normally distributed (*p*s > 0.05). Overall, the study sample met full criteria for a number of psychiatric disorders, including generalized anxiety disorder (32.7%), major depressive disorder (18.4%), post-traumatic stress disorder (18.4%), BPD (12.2%), OCPD (10.2%), social anxiety disorder (10.2%), APD (8.2%), and OCD (6.1%). Descriptive statistics for self-report measures of depression, anxiety, and misophonia symptoms, as well as number of personality disorder symptoms in the high and low misophonia groups are reported in Table 1.

**TABLE 1:** Descriptive statistics for measures of misophonia, anxiety, depression, and number of personality disorder symptoms.

Measures	High misophonia *M (SD)*	Low misophonia *M (SD)*	*t*(47)	Hedges *g* effect size (95% CI)
Misophonia questionnaire	43.05 (7.52)	17.00 (9.99)	−9.67**	2.81 (2.01, 3.61)
Beck anxiety inventory	20.00 (10.08)	11.03 (6.64)	−3.76**	1.09 (0.47, 1.70)
Beck depression inventory	21.74 (12.22)	13.30 (8.83)	−2.80*	0.81 (0.21, 1.41)
Number of SCID-II	11.32 (5.53)	4.62 (3.97)	−4.89**	1.42 (0.77, 2.06)
personality disorder symptoms^∧^				

*p < 0.05, **p < 0.001. ^∧^degrees of freedom for this *t*-test is 46, Personality disorder symptoms represent the total number of symptoms across all personality disorders that were reported on the Structured Clinical Interview for DSM-IV (Axis II Disorders).

### Self-Report

Pearson correlations were conducted to examine the relationship between misophonia and symptoms of psychopathology. These results indicated misophonia symptoms were significantly and positively correlated with symptoms of depression and anxiety (see Table 2). Independent samples *t*-tests indicated a significant difference in BDI and BAI scores between the high (*n* = 19) and low misophonia groups (*n* = 30), such that individuals with high misophonia symptoms reported greater depression and anxiety symptom severity than those low in misophonia symptoms and these effects were large in magnitude (Table 1).

**TABLE 2:** Correlations between misophonia, anxiety, depression, and personality disorder symptoms.

Measures	1	2	3	4
1. Misophonia questionnaire				
2. Beck anxiety inventory	0.55**			
3. Beck depression inventory	0.34*	0.62*		
4. Number of SCID-II personality disorder symptoms	0.56**	0.64**	0.57**	

Personality disorder symptoms represent the total number of symptoms across all personality disorders that were reported on the Structured Clinical Interview for DSM-IV (Axis II Disorders). *p < 0.05; **p < 0.01.

### Clinician Rated

With regard to number of PD symptoms, Pearson correlations indicated misophonia severity was significantly correlated with the number of PD symptoms a participant presented with for APD (*r* = 0.32, *p* = 0.03), OCPD (*r* = 0.37, *p* = 0.01), paranoid PD (*r* = 0.35, *p* = 0.02), schizoid PD (*r* = 0.30, *p* = 0.04), and BPD (*r* = 0.46, *p* = 0.001). Additionally, individuals high in misophonia symptoms reported a greater number of PD symptoms than those low in misophonia (Table 1).

When examining the presence or absence of psychiatric diagnoses, chi-square tests indicated no significant difference between the high vs. low misophonia symptom groups with regard to the presence of a current mood disorder [*X*^2^(1) = 1.04, *p* = 0.31], current anxiety disorder [*X*^2^(1) = 1.50, *p* = 0.22], or current eating disorder [*X*^2^(1) = 0.11, *p* = 0.74]. However, individuals high in misophonia were significantly more likely to meet criteria for a PD compared to those low in misophonia [*X*^2^(1) = 15.12, *p* < 0.001]. With regard to specific PDs, individuals high in misophonia were significantly more likely to meet criteria for APD [*X*^2^(1) = 6.66, *p* = 0.01] and BPD [*X*^2^(1) = 5.49, *p* = 0.02] than those low in misophonia. There were no significant differences between groups with regard to OCPD [*X*^2^(1) = 0.97, *p* = 0.32], paranoid PD [*X*^2^(1) = 1.56, *p* = 0.21], and schizoid PD [*X*^2^(1) = 1.56, *p* = 0.21]. Differences between groups in narcissistic PD, histrionic PD, schizotypal PD, antisocial PD, and dependent PD could not be examined because these diagnoses were not present in the current sample.

### Mediational Analyses

Next, analyses examined whether anxiety and depression symptoms mediated the relationship between symptoms of PDs and misophonia. First, we examined the relationship between misophonia and a set of potential covariates (i.e., age, sex, and race). This set of predictors was not significant [*F*(3,45) = 1.041, *p* = 0.384]. Individual effects showed that age [*B* = −0.132, *t*(45) = −0.512, *p* = 0.611], sex [*B* = −2.038, *t*(45) = −0.281, *p* = 0.780] and race [*B* = −1.467, *t*(45) = −1.601, *p* = 0.116] did not significantly predict misophonia. Therefore, we chose not to include these variables in subsequent analyses.

Next, Model 1 (Figure 1) was examined with anxiety disorder symptoms (BAI) as the mediator. As seen in the figure, PD symptoms were significantly associated with high anxiety, which in turn significantly predicted misophonia symptoms. Additionally, both the direct path and the indirect path (Index of Mediation = 0.23, *SE* = 0.11, Bias Corrected 95% CI: LL = 0.03, UL = 0.45) from PD symptoms to misophonia symptoms was significant; i.e., because 0 is not included in the CI, we can conclude that the indirect effect of PD symptoms on misophonia symptoms through the mediating effect of anxiety is significant. This result suggests anxiety symptoms partially mediated the relationship between PD symptoms and misophonia symptoms.

Model 2 was examined with depression symptoms (BDI) as a mediator. As seen in Figure 2, PD symptoms significantly predicted high levels of depression and the direct path from PD symptoms to misophonia was also significant. However, depression symptoms did not significantly predict misophonia. Furthermore, the indirect path between PD symptoms and misophonia through depression symptoms, was not significant (Index of Mediation = 0.02, *SE* = 0.13, Bias Corrected 95% CI: LL = −0.28, UL = 0.25), suggesting, unlike anxiety, depression did not significantly mediate the relationship between PD and misophonia.

Finally, a parallel mediation model was examined, including both anxiety and depression symptoms as mediators. Similar to the previous models, personality disorder symptoms significantly predicted high anxiety symptoms (*B* = 1.03, *SE* = 0.18, *p* < 0.01) and depression symptoms (*B* = 1.10, *SE* = 0.24, *p* < 0.01). Additionally, high anxiety symptoms predicted increased misophonia (*B* = 0.69, *SE* = 0.29, *p* < 0.05). However, unlike the simple mediation model, depression symptoms significantly predicted misophonia symptoms in the opposite direction (*B* = −0.016, *SE* = 0.22, *p* < 0.05) (i.e., high depression predicted low misophonia, when including anxiety as a mediator). The indirect effects of both anxiety (Index of Mediation = 0.26, *SE* = 0.12, Bias Corrected 95% CI: LL = −0.03, UL = 0.46) and depression (Index of Mediation = 0.02, *SE* = 0.13, Bias Corrected 95% CI: LL = −0.33, UL = 0.24) were no longer significant.

## Discussion

The purpose of this study was to examine associations of misophonia with psychopathology and explore whether anxiety or depression mediated the relationship of misophonia and personality disorders (PDs) using validated semi-structured diagnostic interviews. To our knowledge, this is one of the first studies to use the SCID-I and SCID-II to begin investigating the presence of psychiatric disorders in individuals with misophonia.

As predicted, results indicated more severe misophonia symptoms were associated with higher self-reported symptoms of anxiety and depression, as well as a greater number of PD symptoms. However, counter to predictions, clinician rated measures of psychopathology indicated no difference between individuals with high vs. low misophonia symptoms with regards to a current diagnosis of a mood, anxiety, or eating disorder. One reason for the discrepancy between the dimensional and categorical results may be the small sample size, which limited the ability to detect differences in diagnoses between high and low misophonia groups. On the other hand, individuals high in misophonia were more likely to meet criteria for a PD, specifically avoidant or borderline PD, compared to those low in misophonia.

Overall, these results are consistent with extant literature noting relationships between misophonia and symptoms of psychopathology (e.g., Wu et al., 2014; Schröder et al., 2017; Frank and McKay, 2019; Natalini et al., 2019). The results indicating a relationship between high misophonia symptoms and number of PD symptoms are of particular interest. The use of a dimensional measure of PDs is a strength of this study given the significant overlap in diagnostic criteria among PDs (Krueger, 2005; Zimmermann et al., 2019). The association of misophonia with more PD symptoms may indicate symptom overlap between these conditions or shared vulnerabilities in the development of symptoms. For example, recent research indicates misophonia is positively associated with neuroticism as well as difficulties regulating emotions; these two factors are also implicated in the development and maintenance of personality disorders such as BPD (Sauer-Zavala and Barlow, 2014; Cassiello-Robbins et al., 2020).

To identify whether common symptoms of psychopathology (e.g., anxiety, depression) might underlie the relationship between misophonia and PD symptoms, we conducted three mediational analyses exploring anxiety, depression, and the combination of both as mediators.

The combined analyses did not yield significant effects for both emotions. However, consistent with our hypothesis, when looking at individual emotions, anxiety emerged as a significant mediator between misophonia and PD symptoms. These results are consistent with those noted by Wu et al. (2014) in that anxiety partially mediated the relationship between PD and misophonia symptoms. Taken together, these preliminary findings along with those of Wu et al. (2014) raise the possibility that anxiety may be a primary emotion that needs to be better understood in the assessment and treatment of misophonia. This result begins to raise the question of whether “hatred of sound” is the most appropriate way to characterize misophonia and suggests more research is needed to understand the role of anxiety in the experience of misophonia.

Notably, the PDs that were significantly related to misophonia in this sample (borderline and avoidant) as well as dysregulated anxiety are all characterized by avoidance of stimuli perceived as aversive (e.g., anxiety-provoking stimuli, rejection; Sauer-Zavala and Barlow, 2014). This observation may inform treatment. For example, cognitive behavioral therapies (CBTs) are well-established for the treatment of anxiety and some personality disorders (e.g., BPD; Hofmann and Smits, 2008), and target reductions in avoidance and the acquisition and generalization of skills to respond effectively to emotionally evocative cues. Treatments such as Dialectical Behavior Therapy (Linehan, 2014) specifically target personality disorders symptoms by teaching patients skills for managing strong emotions, including anger, and anxiety, which are also prominent in misophonia. As such, this study contributes to a small but growing literature suggesting CBT-based approaches may be effective for treating individuals with misophonia (Schröder et al., 2017; Frank and McKay, 2019; Cassiello-Robbins et al., 2020). However, existing studies remain preliminary and no randomized trials have been reported for misophonia to date. Thus there is a need for future research to examine the acceptability, feasibility, and efficacy of these approaches for treating symptoms of misophonia.

The results of this study should be considered in light of its limitations. First, similar to other preliminary studies on misophonia, the sample size is relatively small, predominantly female, non-Hispanic, and White. Thus, results should be considered preliminary. Larger studies with more diverse samples examining the association of misophonic symptoms with the full range of psychopathology are needed in order to draw more generalizable conclusions. Second, participants were recruited from a facility specializing in treatment of PDs. Therefore, it is possible the presence of PDs in the current sample is an artifact of the center from which the data were collected. Despite this possibility, our results are in line with other studies indicating a relationship between misophonia and PDs. Third, a limitation of misophonia research in general is the lack of gold-standard assessments. The MQ, which was used in this study, has demonstrated acceptable psychometrics with an undergraduate sample, but has not been validated in clinical or community samples. Therefore, its validity in participants with psychiatric disorders is unknown. Fourth, only a subsample of the clinician-rated interviews were assessed for inter-rater reliability. Thus it is possible rater drift still occurred in this study. Fifth, the mediational analyses conducted used cross-sectional data. Therefore, we were not able to examine how misophonic triggers, anxiety, and PD symptoms interact over time. Future research would benefit from longitudinal data to provide a clearer understanding of how these constructs interact over time and to establish directionality (or lack of) between these variables.

Despite these limitations, the results of this study add important information to the nascent field of misophonia research. Though preliminary, these results highlight the co-occurrence of misophonia with symptoms of personality disorders as well as the potential importance of anxiety in understanding this condition. Future work will benefit from continuing to examine these relationships in order to improve our understanding of misophonia and develop efficacious treatments.

## Data Availability Statement

The datasets generated for this study are available on request to the corresponding author.

## Ethics Statement

The studies involving human participants were reviewed and approved by the Duke University Medical Center IRB. The patients/participants provided their written informed consent to participate in this study.

## Author Contributions

CC-R and DA conducted the statistical analyses. CC-R and MR wrote, revised, and synthesized revisions of the manuscript from other authors. All authors contributed to the intellectual development and writing of the manuscript.

## Conflict of Interest

MR is a scientific advisor for BehaVR and Odin. He also sits on the scientific advisory board for the Misophonia Research Fund. The remaining authors declare that the research was conducted in the absence of any commercial or financial relationships that could be construed as a potential conflict of interest.
